# Innovation and tinkering in the evolution of oxidases

**DOI:** 10.1002/pro.4310

**Published:** 2022-04-26

**Authors:** Jagoda Jabłońska, Dan S. Tawfik

**Affiliations:** ^1^ Department of Biomolecular Sciences Weizmann Institute of Science Rehovot Israel

**Keywords:** enzymes, evolution, oxidases, oxygen, phylogenetics

## Abstract

Although molecular oxygen is a relative newcomer to the biosphere, it has had a profound impact on metabolism. About 700 oxygen‐dependent enzymatic reactions are known, the vast majority of which emerged only after the appearance of oxygen in the biosphere, circa 3 billion years ago. Oxygen was a major driving force for evolutionary innovation—~60% of all known oxygen‐dependent enzyme families emerged as such; that is, the founding ancestor was an O_2_‐dependent enzyme. The other 40% seem to have diverged by tinkering from pre‐existing proteins whose function was not related to oxygen. Here, we focus on the latter. We describe transitions from various enzyme classes, as well as from non‐enzymatic proteins, and we explore these transitions in terms of catalytic chemistry, metabolism, and protein structure. These transitions vary from subtle ones, such as simply repurposing oxidoreductases by replacing an electron acceptor such as NAD by O_2_, to drastic changes in reaction mechanism, such as turning carboxylases and hydrolases into oxidases. The latter is more common and can occur with strikingly minor changes, for example, only one mutation in the active site. We further suggest that engineering enzymes to harness the extraordinary reactivity of oxygen may yield higher catabolic power and versatility.

## 1 BACKGROUND

Molecular oxygen (O_2_ or oxygen hereafter) is a fascinating molecule. It appeared on our planet relatively late, and its emergence was a pivotal event that shaped the biosphere as we know it today. Life emerged under a reducing atmosphere, and the Last Universal Common Ancestor (LUCA) was most certainly a strict anaerobe.^1^ Nevertheless, oxygen was perhaps not alien to the LUCA. It might have been produced intracellularly—indeed, enzymes that neutralize toxic oxygen by‐products seem to date back to the LUCA^1^, ^2^—but was not part of primary metabolism. Atmospheric oxygen is a by‐product of the oxygenic photosynthesis that evolved in the ancestors of contemporary Cyanobacteria and became widely available in the biosphere shortly thereafter.^2^, ^3^ It took, however, >0.5 BY for oxygen to permanently accumulate in the atmosphere, an event called the Great Oxidation Event (GOE) that occurred about 2.4 BYA. Molecular oxygen spawned a burst of emergence of oxygen‐dependent enzymes (hereafter, O_2_ enzymes/oxidases) that may have initially served to mitigate its potential toxicity but also took advantage of the free energy available from using O_2_ as an electron acceptor.

Oxygen‐dependent enzymes provide simple yet powerful tools for studying the impact of oxygen on proteomes and, in turn, on organismal phenotypes.^4^ The evolutionary history of these enzymes also serves as a proxy for dating the appearance of oxygen in the biosphere.^2^ To this end, we listed all the known O_2_‐dependent enzymes found in KEGG^5^ and assigned them to 136 protein families (using the Pfam protein families categorization^6^). In 81 of these families, the primary function is related to oxygen, as indicated by nearly all family members being O_2_ enzymes. By parsimony, this means that the founders (the earliest ancestors) of these families were O_2_ enzymes (*O*_*2*_‐*founding* families). In other words, these families emerged de novo in the context of oxygen. In the other 55 families, however, the O_2_ function is sporadic (i.e., the function of most family members is unrelated to oxygen). Therefore, the *niche* families contain both O_2_‐dependent and O_2_‐independent enzymes with high sequence identity between these two types of members. Since the non‐O_2_ function in those families is dominant, the O_2_‐dependent family members likely evolved from a non‐O_2_ ancestor. In principle, for *niche* families, one can track down the functional transitions that occurred within the family, identify the ancestral function, and describe how the O_2_ enzyme(s) diverged from a non‐O_2_ predecessor. As such, they provide an opportunity for understanding how an O_2_ enzyme evolves. Individual cases of oxygen‐dependent enzymes emerging from non‐O_2_ ancestors have been described.^7^, ^8^, ^9^ Here, we present a broader picture of this phenomenon, highlighting the innovation brought about by oxygen to the enzymatic world on two levels: (i) enzyme mechanism and (ii) metabolic context. We also provide insights into protein structure changes that drove those transitions. Our analysis also highlights specific aspects of oxygen‐dependent enzymes' nature that could be of significance in the enzyme engineering field, in particular, engineering hydrolases into oxidases.

## 2 RESULTS

### 2.1 Transitions in enzymatic functions

About 60% of all known enzyme families (as classified by Protein Structure Classification database CATH) contain only enzymes belonging to the same reaction class, that is, the entire family shares the same first digit of an Enzyme Commission (EC) class; for example, oxidoreductases (EC 1.‐.‐.‐), the EC class to which O_2_‐utilizing enzymes, in general, belong.^11^ This means that these families did not drastically diverge with respect to the enzymatic mechanism in the course of evolution, preserving the overall catalytic chemistry accommodating alternative substrates. Conversely, if a family spans the members belonging to multiple EC classes (i.e., hydrolases and oxidoreductases), more fundamental transitions that regard the catalytic chemistry have occurred.

On the metabolic level, oxygen drove the emergence and expansion of hundreds of reactions and pathways.^12^ The O_2_‐dependent reactions could have emerged de novo with the oxygenated biosphere; thus, neither the substrates nor the products exist in an O_2_‐independent context. Otherwise, the alternative O_2_‐free reaction involving the same reactants existed prior to biosphere oxygenation and was later adapted to utilize oxygen as an electron acceptor.

In the following chapters, we will systematically discuss the transitions in enzymatic mechanisms and metabolic repertoire that came with the onset of molecular oxygen, as well as explore the local and global structural hotspots for these transitions.

### 2.2 The divergence of chemistry (enzyme mechanism)

**FIGURE 1:**
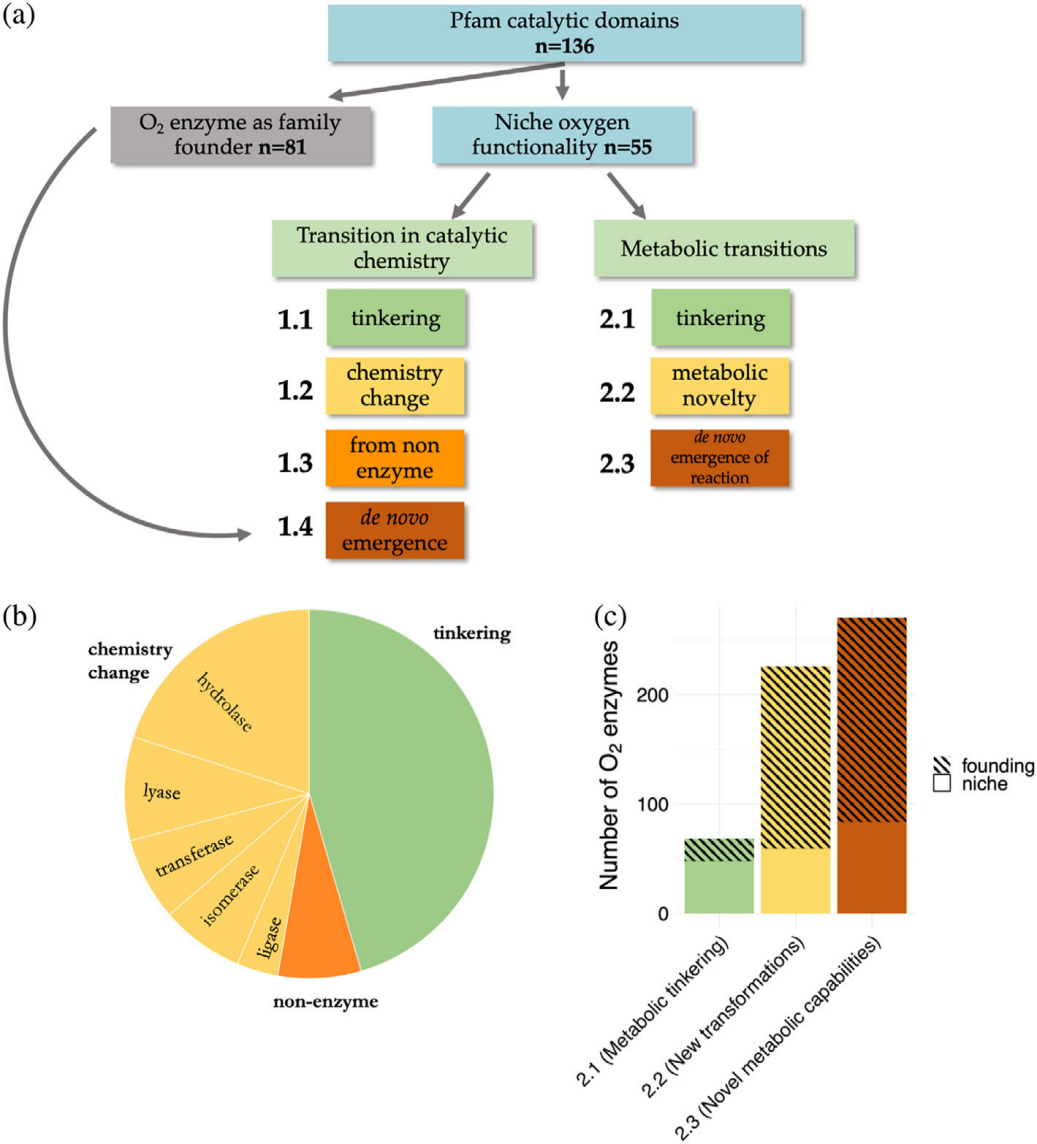
Modes of the evolution of O_2_ enzymes. (a) Analysis pipeline. All O_2_ enzymes (EC classes) that have known sequences in the ExPASy enzyme database were identified, then classified their catalytic domains to Pfam families (blue) as detailed in the Supplementary Information. The O_2_ enzyme‐containing families were then divided into *niche* and founding categories. The founding families (gray) represent emergences de novo of an O_2_ enzyme, and their evolutionary origin and mode of emergence cannot be tracked down.^2^ The remaining 55 families (dubbed *niche* families) represent cases of divergence of an O_2_ enzyme from a founder whose function is unrelated to O_2_ (Table S1). In these cases, the ancestral function can be inferred (by examining the non‐O_2_ family members), and the mode of divergence of the O_2_ enzyme can be tracked down. The *niche* families were analyzed for changes in the catalytic chemistry and the degree of a metabolic innovation in relation to their non‐O_2_ ancestor. The categorization into *niche* and founding was adopted from Reference 2 with the addition of two Pfam families that represent pyridoxal‐dependent enzymes (PLP enzymes). These families have been shown to contain O_2_‐dependent enzymes,^10^ but these enzymes were not detected in our initial analysis because they do not have any EC number assigned. (b) The frequency of Pfam families with redox (green), non‐O_2,_ non‐redox enzyme (yellow), or non‐enzymatic protein (orange) as a family founder in families with O_2_‐dependent members. The founding activity of the family has been assigned by parsimony (see text). (c) The frequencies of metabolic transitions within founding and *niche* families. The *y*‐axis is the number of O_2_ enzymes (O_2_‐dependent EC classes), and *x*‐axis is the category of metabolic transition

When examining evolutionary transitions between family members, the degree of change in the catalytic chemistry, that is, differences in the nature of the catalyzed reactions and the mode by which these reactions are catalyzed by the original and the newly diverged enzyme, can be divided into three major categories. The first and least drastic is “tinkering,” which encompasses transitions from enzymes that perform oxidations with various electron acceptors other than O_2_, such as NAD^+^, to enzymes that use O_2_ as an acceptor (Figure 1a, category 1.1). This sort of transition is associated with the preservation of the first EC digit. In the second level of innovation, the non‐O_2_ progenitor catalyzes a non‐redox reaction(s), sometimes with a completely unrelated catalytic chemistry—consequently, the first EC digit changes. Curiously, changes in electron acceptor and changes in enzyme chemistry seem to be nearly equally common. The third level regards transitions from a non‐enzymatic protein to an O_2_ enzyme. Here, a pre‐existing protein scaffold, and sometimes a pre‐existing ligand‐binding pocket, serve as a starting point for a new active site that catalyzes O_2_‐dependent oxidation. The O_2_‐founding families comprise the fourth level of innovation, namely de novo emergence of an O_2_ enzyme, where even the protein scaffold has no detectable oxygen‐independent origin, that is, no O_2_‐independent members in the family.

However, it should be noted that the close homology of an oxidase to another enzyme/protein does not necessarily indicate the ancestry of the latter. The inference is by parsimony, namely by the majority rule—if a family is dominated by enzymes with a given non‐O_2_ function, the ancestor is presumed to have possessed this non‐O_2_ function. However, the alternative scenario, namely the ancestor being an O_2_ enzyme, and this function being lost in most contemporary members, is also possible. To elucidate the directionality of evolution and infer the putative activity of a family ancestors, one would need reliable protein trees of its members. However, such an endeavor is challenging since the accurate functional assignment of family members that show close homology (i.e., separating the oxidases from the other family members) is difficult, if not impossible, in many cases. Nevertheless, in some cases, given the abundance of another activity (many family members have non‐oxygen‐related primary activities), there is little doubt that the oxidase came later (e.g., Metallo‐β‐lactamases).

### 2.3 Tinkering: Transitions within redox enzymes (category 1.1)

As noted before,^11^ most transitions in oxidoreductases occur within the oxidoreductase class (EC 1.‐.‐.‐). Indeed, in 25 of 55 niche families, the oxidases seem to have emerged from another redox enzyme (Figure 1b). The transitions involve the replacement of the electron acceptor from, for example, NAD(P)^+^ (dehydrogenase).^13^ For instance, sulfite dehydrogenase and sulfite oxidase both belong to the molybdopterin‐dependent enzymes family (PF00174). They perform essentially the same reaction with different electron acceptors. Similarly, the large and diverse GMC oxidoreductase family (PF05199) includes choline and cellobiose dehydrogenases, as well as methanol, glucose, and pyranose oxygenases.^14^ As the electron acceptor, GMC oxidoreductases can employ O_2_ or alternative electron acceptors such as quinones, phenol radicals, or metal ions.

**FIGURE 2:**
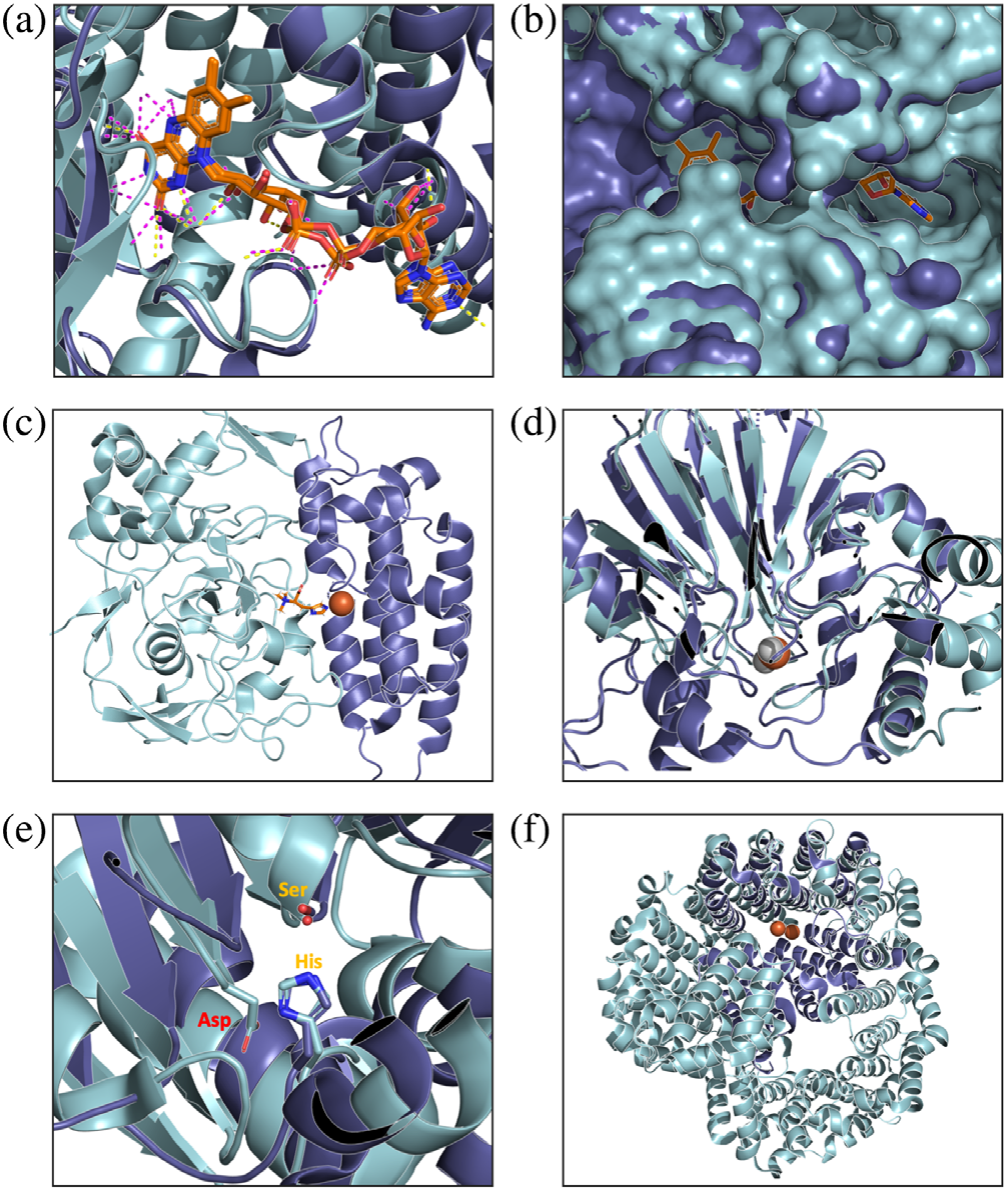
Examples of structural transitions of oxidases. All oxygen‐dependent enzymes are colored purple, their oxygen‐independent counterparts blue, and substrates and cofactors orange. (a) Acyl‐CoA oxidase (PDB: 1IS2) and dehydrogenase (PDB: 3MDD). The oxygen dependency seems to be dictated by the reduced hydrogen bonding with FAD (magenta and yellow dashed lines). (b) Xanthine oxidase (PDB: 1FIQ) and dehydrogenase (PDB: 1F04). The dislocation of the active site loop blocks the NAD^+^ from accessing the oxidase active site. (c) Sulfatase‐modifying factor (PDB: 4X8E). The oxidase active site is formed on the interface of two non‐oxidase domains. (d) Persulfide dioxygenase (PDB: 4YSL) and glyoxalase II (hydrolase, PDB: 1QH5). The hydrolase to oxidase transition likely occurred by the loss of one of two hydrolase active site metal ions (gray) and replacement with a water molecule. E, 1‐H‐3‐hydroxy‐4‐oxoquinaldine 2,4‐dioxygenase (PDB: 2WM2) and thermophilic esterase (PDB: 4UHH). Dioxygenase utilizes catalytic dyad (orange) instead of a triad (orange and red) characteristic for hydrolases from this family. (f) Deoxyhypusine monooxygenase (PDB: 4D4Z) and importin β (PDB: 3ND2). Evolution of oxidase from non‐enzyme (importin β) via dramatic structural rearrangements

The structural differences between dehydrogenases and oxidases belonging to the same family tend to be subtle. For example, in the acyl‐CoA oxidase/dehydrogenase family, the oxygen dependency seems to be dictated by the reduced hydrogen bonding with FAD that served as a cofactor in oxidase, making the active site more solvent‐accessible (Figure 2a).^15^ Indeed, some enzymes show acceptor plasticity, for example, xanthine oxidase/ dehydrogenase, where the same protein can utilize O_2_ or NAD^+^ and the interconversion is dictated by dislocation of the active site loop that blocks the access of NAD^+^ to the FAD cofactor in the oxidase^16^, ^17^ (Figure 2b). These bifunctional enzymes represent an evolutionary intermediate between a dehydrogenase and an oxidase, indicating how readily such a transition can occur.

Note that in some cases, the reaction catalyzed by the oxidase and the related oxygen‐independent oxidoreductase remains the same with respect to the primary substrate and product, that is, only the electron acceptor is replaced (e.g., sulfite dehydrogenase/oxidase that both catalyze the oxidation of sulfite to sulfate with ferricytochrome c and oxygen as an electron acceptor, respectively).^13^ Such transitions are primarily driven by the large thermodynamic gain associated with the use of O_2_ as an acceptor (as discussed in Metabolic transitions below).

However, in most cases, the substrate and/or the reaction product have changed along with the electron acceptor. Namely, the closest O_2_‐ and non‐O_2_ homologs (sequence‐wise) use a different substrate and yield a different product (in addition to using a different electron acceptor). Plausibly, the original dehydrogenase activity was not retained because the equivalent oxidase proved far more efficient and took over. Alternatively, the O_2_ enzyme may have diverged independently of the non‐O_2_ analog. Across the enzyme world, homologs (evolutionarily related enzymes) are as common as analogs (enzymes of unrelated origins that catalyze the same reaction),^18^ and this trend is also seen with respect to dehydrogenase/oxidase pairs. Glucose oxidase (EC 1.1.3.4), for example, is a Rossmann fold oxidase. Its closest non‐O_2_ homolog is choline dehydrogenase (EC 1.1.99.1). The analogous non‐O_2_ equivalent of the glucose oxidase, a ubiquinone‐dependent glucose dehydrogenase (EC 1.1.5.2), belongs to the beta‐propeller clan and shares no homology to the O_2_ enzyme. Overall, an entire spectrum can be seen with respect to the divergence of electron acceptors, from the very same enzyme using different acceptors to unrelated emergences; representative examples of these scenarios are shown in Table 1.

**TABLE 1:** Examples of O_2_ and non‐O_2_ reaction pairs divided into three categories: (1) enzymes with dual O_2_ and O_2_‐free activity; (2) homologous enzymes belonging to the same Pfam family, products of different genes; (3) non‐homologous enzymes belonging to different Pfam families (and clans)

Category	O_2_ enzyme name and EC	O_2_ Pfam	Non‐O_2_ enzyme name and EC	Non‐O_2_ Pfam
Electron acceptor change (dual activity)	Xanthine oxidase (1.17.3.2)	PF01315﻿ (no clan)	Xanthine dehydrogenase (1.17.1.4)	PF01315 (no clan)
	Cellobiose oxidase (1.1.99.18)	PF00732﻿ (CL0063)	Cellobiose dehydrogenase (1.1.99.18)	PF00732﻿ (CL0063)
	Dihydroorotate oxidase (1.3.3.1)	PF01180 (CL0036)	Dihydroorotate dehydrogenase (1.3.99.11)	PF01180 (CL0036)
Electron acceptor change (the same Pfam family)	Acyl‐CoA oxidase (1.3.3.6)	PF08028 (CL0087)	Acyl‐CoA dehydrogenase (1.3.8.1)	PF08028 (CL0087)
	Choline oxidase (1.1.3.17)	PF00732﻿ (CL0063)	Choline dehydrogenase (1.1.99.1)	PF00732 (CL0063)
	Sarcosine oxidase (1.5.3.1)	PF01266 (CL0063)	Sarcosine dehydrogenase (1.5.8.3)	PF01266 (CL0063)
	Dimethylglycine oxidase (1.5.3.10)	PF01266 (CL0063)	Dimethylglycine dehydrogenase (1.5.8.4)	PF01266 (CL0063)
Different Pfam families	Glycerol‐3‐phosphate oxidase (1.1.3.21)	PF01266 (CL0063)	Glycerol‐3‐phosphate dehydrogenase (1.1.1.8)	PF07479﻿ (CL0106)
	Glycolate oxidase (1.1.3.15)	PF01070 (CL0036)	Glycolate dehydrogenase/reductase (1.1.99.14/1.1.1.26)	PF02913 (CL0277)/PF00389 (CL0325)
	Glucose oxidase (1.1.3.4)	PF00732﻿ (CL0063)	Glucose dehydrogenase (1.1.5.2)	PF01011﻿ (CL0186)
	L‐amino acid oxidase (1.4.3.2)	PF01593﻿ (CL0063)	L‐amino acid dehydrogenase (1.4.3.5)	PF01243﻿ (CL0336)
	Coproporphyrinogen oxidase (1.3.3.3)	PF01218 (no clan)	Coproporphyrinogen dehydrogenase (1.3.98.3)	PF04055 (CL0036)
	Oxidative cyclase (AcsF) (1.14.13.81)	PF02915 (CL0044)	O_2_‐independent oxidative cyclase (BchE) (1.21.98.3)	PF04055﻿ (CL0036)

### 2.4 Transitions from non‐redox enzymes (category 1.2)

The next innovation level involves more fundamental changes in catalyzed chemistry and, accordingly, changes in the reaction mechanism. Such transitions are reflected in changes in the first EC digit. We identified niche O_2_ enzymes showing close homology to enzymes belonging to 5 of the remaining 6 EC classes (i.e., all classes except the oxidoreductases class; Figure 1b, Table S1).

Interestingly, the most common progenitors of O_2_ enzymes are hydrolases (EC 3.‐.‐.‐; 11 of 48 families). Numerous oxidases can be identified in the two major hydrolase superfamilies—the Metallo‐β‐lactamases and the alpha/beta hydrolases. Given the dominance of the hydrolase activity in these superfamilies and their early pre‐LUCA origin, there is little doubt that the oxidases diverged from a pre‐existing hydrolase. Most of these oxidases make use of a metal ion cofactor. However, cofactor‐independent oxidases^19^ such as PqqC, urate oxidase, coproporphyrinogen oxidase, and Renilla luciferase have also diverged from hydrolases. It appears that oxygenation has initially evolved as a side reaction of the original hydrolytic activity, and the enzyme was eventually turned into a specialized oxidase. Accordingly, although the change in the type of the catalyzed reaction is drastic, it seems that the hydrolase‐oxidase transition can be readily achieved via minor changes in the active site and/or via changes in the active site metal ion composition (see “Small (mutational) steps induce big (chemical) changes”). We suggest that this mode of divergence can be implemented in the laboratory with some practical applications, as elaborated in the Concluding Remarks section.

Other unexpected transitions to oxidases have been documented, for example of PLP‐dependent decarboxylases, whose O_2_ relatives have been shown to perform an oxidative decarboxylation using PLP as a cofactor.^10^, ^20^ Other examples include sugar isomerases from the Cupin clan that were shown to be ancestors of peptide‐modifying oxidases (i.e., wybutosine hydroxylase).^7^

### 2.5 From a nonenzyme to an oxidase (category 1.3)

The third level in the innovation spectrum regards the evolution of oxidases from non‐enzymatic precursors. A novel enzyme can evolve from scratch, for example, from a ligand binder.^21^, ^22^ Sulfatase‐modifying factor (EC 1.8.3.7) is a two‐domain protein, in which one domain originated from a hydrolase, while the other originated from a lectin (a carbohydrate‐binding protein). The newly formed oxidase active site resides at the interface between these two domains (unlike the cases described in the above section, the active site of the original hydrolase does not overlap with the oxidase, Figure 2c).

Another example is deoxyhypusine monooxygenase (EC 1.14.99.29). It belongs to the HEAT repeat family of tandemly repeated helical domains. Proteins in this and other families in the clan mediate protein–protein interactions in diverse contexts, for example, a scaffolding subunit of the human a subunit of protein phosphatase.^23^ The number and arrangement of the HEAT repeats, as well as binding of catalytic iron, seem to mediate the de novo emergence of an O_2_ active site from non‐O_2_ protein–protein interaction module (see “Structural (fold) changes”).

### 2.6 Metabolic transitions

Oxygen drove a significant expansion of metabolism. As shown here and before, its availability in the biosphere to begin with and later in the atmosphere is estimated to have led to the emergence of nearly 600 new metabolic reactions and 650 metabolites.^12^ However, as discussed above, many of the transformations catalyzed by O_2_ enzymes have an analogous O_2_‐independent counterpart (e.g., dehydrogenases that use an alternative electron acceptor to mediate the same oxidation; Table 1). Thus, to assess the degree of metabolic innovation that accompanied the emergence of O_2_ enzymes, for the 565 identified O_2_ enzymatic reactions, we looked for alternative enzymatic transformations (or absence thereof) that synthesize/catabolize the exact product/substrate in an O_2_‐independent manner. We accordingly defined three levels of metabolic innovation (Figure 1).

In cases where the substrate, the product, and their interconversion are all associated with a particular non‐O_2_ transformation, we assumed that the latter preceded the emergence of the O_2_ enzyme. The O_2_ enzyme has only allowed the very same transformation to occur more efficiently—a scenario we dubbed *metabolic tinkering* (2.1). In the second level, although the O_2_‐mediated transformation has no non‐O_2_ analog, either the substrate (in catabolic reactions) or the product (in anabolic reactions) of the O_2_ enzyme is produced by alternative O_2_‐independent reactions (*new transformations*, 2.2). In cases that present the highest degree of metabolic innovation (*novel metabolic capabilities*, 2.3), we could not detect alternative non‐O_2_ reactions leading to the synthesis or degradation of the substrate/product of the O_2_ enzyme. The results of this analysis are summarized in Figure 1C, where the three levels of metabolic innovation are shown for both O_2_‐niche and O_2_‐founding enzymes.

### 2.7 Metabolic tinkering (category 2.1)

As noted above, some oxidation reactions can be performed in an oxygen‐free manner. A total of 19 such analogous reactions have been identified before.^13^ We identified 48 additional ones (Figure 1C, Table S1). As expected, this metabolic tinkering is more prevalent in the O_2_‐niche families. Foremost, oxidoreductases tend to change their electron acceptor (see the above section on Category 1.1 and Table 1). Conversely, as discussed below, founding families, where the family progenitor was likely an O_2_ enzyme, are more often associated with de novo metabolic emergences.

**FIGURE 3:**
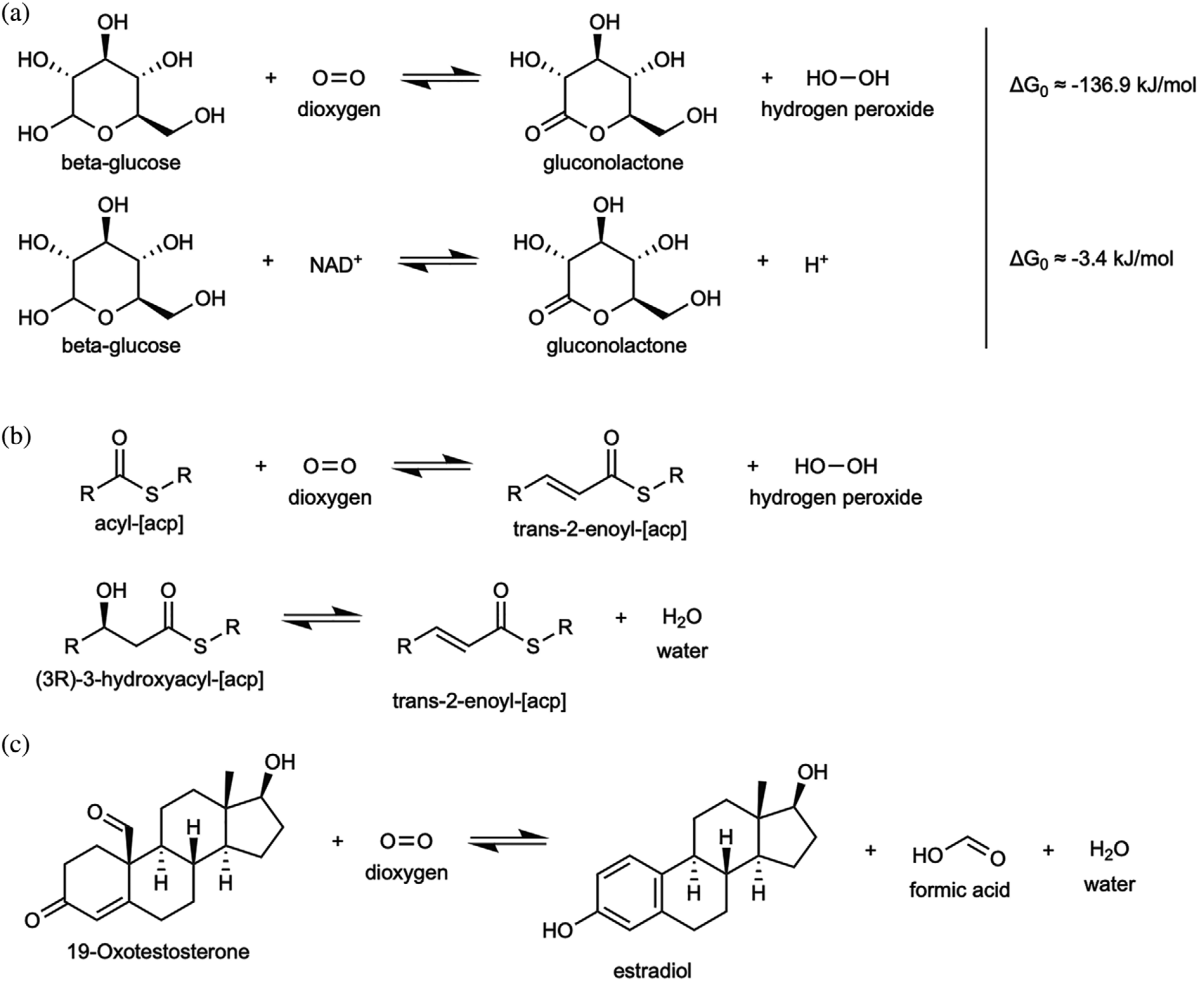
Example reactions representing three types of metabolic transitions of oxygen enzymes. (a) Metabolic tinkering of glucose oxidase (EC 1.1.3.4) to glucose dehydrogenase (EC 1.1.5.2), where both substrates and products are retained and only the electron acceptor changes. The oxygen‐dependent reaction is much more favorable thermodynamically (based on lower Δ*G*_0_). (b) Novel transformation in the biosynthesis of unsaturated fatty acids, where the product of the oxygen‐dependent reaction remained the same, but all the other reactants changed. (c) De novo reaction emergence. There is no alternative, oxygen‐free reaction involving the primary substrates and products, here 19‐Oxotestosterone and estradiol

The thermodynamic drive is a major player in metabolic tinkering. The oxidation potential of NAD^+^, or FAD, is in the same range, or even lower, for driving the oxidation of certain metabolites. Thus, O_2_‐driven reactions occur with highly favorable thermodynamics compared to their non‐O_2_ counterparts. For example, glucose oxidase (EC 1.1.3.4) has ΔG^0^ ≈ −136.9 kJ/mol compared to −3.4 kJ/mol for the glucose dehydrogenase (EC 1.1.1.47, Figure 3a) or –155.3 for xanthine oxidase (EC 1.17.3.2) and –25.9 for xanthine dehydrogenase (EC 1.17.1.4).^24^ A low free energy gap (ΔG^0^ ≈ 0) means reversibility, hence a low net forward flux, thus demanding high enzyme concentrations or extremely efficient enzymes to maintain sufficient flux.^25^ Oxygenations (O_2_‐mediated oxidations) are, in effect irreversible, thereby allowing high flux at low enzyme concentrations.

### 2.8 New transformations (Category 2.2)

In some cases, however, even though an equivalent O_2_‐free reaction with identical substrates and products does not exist, there is an alternative reaction involving the same substrate(s) or product(s). For synthetic anabolic transformations (as annotated in KEGG^5^), whose primary outcome is a given product, we identified all single‐step transformations that can synthesize the same product in an O_2_‐independent manner (obviously using a different substrate than the one used by the O_2_ enzyme). Similarly, for catabolic transformations whose primary function is the degradation of a given substrate, we searched for all single‐step transformations that can transform the same substrate (although their product(s) differ from the one produced by the corresponding O_2_ enzyme). We identified 166 such analogous reactions in founders and 60 in niches (Table S2). The presence of alternative reactions suggests that the substrate/product of the O_2_‐dependent reaction existed prior to the emergence of the O_2_ enzyme. The latter, however, allowed a faster (as above) and/or more efficient way (e.g., fewer steps in a pathway or synthesis from more readily available precursors) of producing a certain metabolite and of catabolizing others.

With respect to anabolism, the typical example is the biosynthesis of unsaturated fatty acids, which can be achieved in an O_2_‐dependent manner through both aerobic and anaerobic desaturation (Figure 3b). The aerobic pathway, being utilized by both prokaryotes and eukaryotes, is much more phylogenetically widespread.^26^ In catabolism, oxygen plays a significant role in the degradation of heme, a prosthetic group of multitudes of essential proteins. Out of seven heme degradation pathways, only one of them (called “heme degradation pathway V” in MetaCyc) does not require oxygen,^27^ and the heme oxygenase reaction is performed by a SAM‐dependent anaerobilin synthase that degrades protoheme to iron and anaerobilin intermediate (Figure 3b). All in all, the introduction of oxygen to the metabolic repertoire of organisms opened alternative, often more efficient routes for catabolizing/anabolizing given compound.

### 2.9 De novo reactions emergence (Category 2.3)

In total, almost half of all O_2_ reactions emerged de novo (270/563), meaning there is no alternative reaction leading to synthesis/degradation of the metabolite produced/used by the O_2_ enzyme. Thus, the emerging O_2_ enzyme opened the door to completely new metabolic capabilities. Both the founding and *niche* families are represented in this category, yet the former dominate it (in contrast to metabolic tinkering, Category 2.1; Figure 1c). It appears, therefore, that in the expansion of metabolic networks, the appearance of new enzymes (emergence of a new protein scaffold and active site) is correlated with the appearance of new metabolites and transformations.^12^, ^28^

Novel anabolic capabilities that are associated with O_2_ are exemplified by steroids that are a hallmark of O_2_‐dependent metabolism since their biosynthesis is strictly dependent on oxygen.^29^ In secondary metabolism, many antibiotics are synthesized exclusively with the use of oxygen (Figure 3c).^30^

The most prominent example regarding novel metabolic capabilities is the degradation of aromatic compounds, including aromatic amino acids. Breaking aromatic rings requires a strong oxidant to overcome the resonance energy that stabilizes these rings.^31^ Accordingly, only a few O_2_‐independent aromatic degradation pathways have so far been identified, and this pathway is known to be an inefficient multistep process.^32^, ^33^ Indeed, it appears that the most immediate impact of the appearance of O_2_ in the biosphere had been increased catabolic capabilities. We have previously mapped 22 O_2_ enzyme families whose emergence seems to mark the appearance of O_2_ in the biosphere about 3 BYA. Most of these early emerging O_2_ enzymes mediate catabolism, including the degradation of aromatics, lipids, and sterols (whose breakdown is kinetically demanding) or of lysine. This suggests that the catabolic potential of O_2_ can also be harnessed for the biodegradation of xenobiotics.

### 2.10 Small (mutational) steps induce big (chemical) changes

Most emergences of O_2_ enzymes involve a novel protein scaffold and active site (O_2_ founding families comprise ~60% of all emergences). Nonetheless, there are multiple examples of an O_2_‐utilizing active site emerging by tinkering, by minor modifications of a pre‐existing active site with no relation to oxygen. Most intriguing are changes that are subtle and may boil down to a single active site residue.^34^

The structural changes needed to repurpose a dehydrogenase to use O_2_ as an acceptor are usually subtle.^15^, ^17^ However, beyond the enhancement of pre‐existing usage of O_2_ as electron acceptor,^35^, ^36^ as far as we could track down, introducing an O_2_‐utilizing capability de novo has not been reported so far (by protein engineering or directed evolution).

Transitions from an active site that catalyzes a non‐redox reaction can be achieved in various ways, most notably via a change in the catalytic metal ion. Alterations in the catalytic metal ion and its ligating environment often drive the divergence of new enzymatic functions.^37^ This is also the case with the divergence of O_2_ enzymes, as exemplified in the above‐mentioned Metallo‐β‐lactamases and cupin sugar isomerases. For cupins, it was implied that the sugar isomerase active site was exapted for catalysis of oxygenation putatively via the binding of an oxygen molecule by the catalytic metal ion that mimics the two oxygen molecules of the enediol intermediate of the sugar isomerases.^7^ Another example, the HD family, is dominated by diverse phosphohydrolases with mono‐ or dinuclear metal centers. The oxidases from this family catalyze the oxidative cleavage of C–C and C–P bonds, especially in organophosphonates.^38^ Unlike the hydrolases that accept a wide range of transition metals, the oxidases seem to all have a diiron metal center. Another example is persulfide dioxygenase (EC: 1.13.11.18), which likely evolved from a hydrolase by the loss of one of the two metal ions and its replacement with a water molecule (Figure 2d). Additionally, the metal ion in the oxidase is iron, as opposed to the dizinc catalytic center seen in most other Metallo‐β‐lactamases (although zinc‐iron combinations are also seen among hydrolases).^8^

Most oxidases are metalloenzymes or use a metal‐containing cofactor such as heme. Nonetheless, cases of non‐metalloenzymes that diverged into oxidases are known. Among these are PLP‐enzymes^10^ and hydrolases. Furthermore, in the case of hydrolases, the catalytic machinery seemed to barely change. For example, enzymes belonging to the alpha/beta hydrolases clan uses a catalytic triad comprised a nucleophile (e.g., serine), histidine, and an acidic residue. At least two oxidases belonging to this clan could be identified: 1‐H‐3‐hydroxy‐4‐oxoquinaldine 2,4‐dioxygenase (HOD) and its close relative 1‐H‐3‐hydroxy‐4‐oxoquinoline 2,4‐dioxygenase (QDO) (Figure 2e). These enzymes that degrade N‐heteroaromatic compounds have neither a catalytic metal ion nor any other cofactor. The closest hydrolases employ a non‐nucleophilic general‐base mechanism, with the catalytic dyad instead of a triad in hydrolases.^15^ Although the mechanism is not entirely clear, it appears that the “oxyanion hole” of the α/β‐hydrolase fold, typically employed to stabilize the oxyanionic tetrahedral intermediate in ester hydrolysis reactions, is also utilized by the oxidases.^9^

All those examples show that in order to significantly change the chemistry of an enzyme, local changes in the active site seem to be sufficient. Indeed, this phenomenon has been noticed before in other enzyme classes. However, for oxygen enzymes, such a small alteration in the active site often comes with a significant thermodynamic boost.

### 2.11 What makes an O_2_ ‐dependent active site?

The electrostatic and structural properties of known oxidases' active sites shed light on the molecular architectures of the oxygen‐dependent active sites. For example, protein positive charges have been identified in the active sites of glucose oxidase, sarcosine oxidase, N‐methyltryptophan oxidase, and fructosamine oxidase. They electrostatically stabilize the transition state for the initial single electron transfer.^39^ Additionally, any electrostatic effect on the activation of O_2_ would be maximized in a non‐polar, desolvated environment rather than in a more polar environment.^40^ On the structural level, smaller active site cavities can sequester the oxygen molecule and yield more kinetically favorable substrate‐active site binding.^41^ It has also been shown that oxygen travels to the oxidase active site through the gas diffusion channels that are gated by conformationally flexible “gating residues” at the direct interface with the active site.^42^ Such gates can be observed, for example, in vanillyl alcohol oxidase, cholesterol oxidase, and persulfide oxidase.^8^, ^43^ Systematic understanding of the building blocks of the oxygen‐dependent active site could aid the efficient enzymes' engineering efforts.

### 2.12 Structural (fold) changes

Active site tinkering, as described above, that leads to chemistry changes, is a common evolutionary scenario. However, in rare cases, global rearrangements of the fold lead to the emergence of the new oxygen‐dependent enzyme from a precursor.

One of such drastic structural changes is mentioned earlier (see “From a non‐enzyme to an oxidase”) deoxyhypusine monooxygenase (EC 1.14.99.29), belonging to the HEAT repeats family (Figure 2f). The family is dominated by non‐enzymatic proteins that mediate protein–protein interactions, transport or have regulatory function.^44^ The oxidase and non‐enzymatic members of the family differ significantly in the number and orientation of the HEAT repeats. The oxidase active site is sandwiched between two layers of repeats. In contrast, other members vary in the orientation and number of the superhelical elements, oftentimes showing elaborate conformations (e.g., importin β, Figure 2f). The conformational change to the shell‐like structure of oxidase was reported to be mediated by the catalytic iron.^23^

## 3 CONCLUDING REMARKS

Oxygen brought about a true revolution in the enzyme world. Its huge thermodynamic advantage catalyzed innovation on multiple levels, including chemistry, metabolism, and protein structure. Looking at those changes through the lenses of oxygen‐dependent enzymes evolution could bring insight into how O_2_ shaped the metabolism and shed light on how modern O_2_ enzymes evolved from the pre‐O_2_ ancestors.

The oxygen emergence of Earth was probably an important factor guiding the evolution of oxygen enzymes. On all three innovation levels, oxygen emergence or GOE might have played a role, being a trigger for both de novo emergence and tinkering of chemistries, metabolism, and protein structures.

Chemistry‐wise, oxidases most frequently evolve from another oxidoreductase by the change of electron acceptor. Nevertheless, multiple O_2_ enzymes can be found in families dominated by hydrolases, which seems to be a common evolutionary trend with potential biotechnological applications. The most prominent of such applications would be engineering new enzymes that utilize oxygen as an electron acceptor to increase their enzymatic efficiency. One must bear in mind, though, that the ancestry is assigned based on the majority rule, where the family ancestor's function is the one dominating the family. For specific cases, a detailed phylogenetic analysis must be carried out.

On the metabolic level, oxygen enabled reactions that otherwise are thermodynamically unfavorable. One of the most prominent examples of metabolic innovation is enzymes degrading aromatic compounds, such as soil pollutants and fertilizers. Soil bacteria, by evolving enzymes that could break the aromatic ring in one or two steps, can grow on the carbon from the aromatic ring.^45^

The innovation in enzyme mechanism and metabolism are intertwined. The founding enzymes dominate in the novel metabolic capabilities (2.3) category, suggesting that not only the enzymatic scaffold but also the reaction the enzyme performs emerged de novo with oxygen. Similarly, enzymatic chemistry tinkering is correlated with metabolic tinkering—the reactions of oxidases that descended from oxidoreductases often have the O_2_‐free counterparts with an alternative electron acceptor.

On the protein level, oxygen brought both small changes in the active site of the precursors, allowing accommodation of the O_2_ molecule by a handful of mutations (active site tinkering) and global structural changes with de novo emergences of active sites and completely new protein families. The active site tinkering examples are eminently tantalizing because in principle, one could evolve oxidase easily from the starting point, the ancestral enzyme. By minor tweaks in the active site, the enzyme could be exapted to utilize oxygen. It is an encouraging observation and a peculiar recipe for efficient enzyme engineering. Designing an enzyme with a versatile hydrolase scaffold and the oxygen‐dependent active site would come with a large thermodynamic gain and, consequently, large product yield.

## AUTHOR CONTRIBUTIONS

**Jagoda Jabłońska:** Conceptualization (equal); formal analysis (equal); investigation (equal); visualization (lead); writing – original draft (equal); writing – review and editing (equal). **Prof. Dan Tawfik:** Conceptualization (equal); formal analysis (equal); investigation (equal); visualization (equal); writing – original draft (equal); writing – review and editing (equal).

## Supporting information


**Appendix S1** Supporting Information.

**Table S1** Pfam families where oxygen‐dependence is a niche function (clarifying comments below)

**Table S2** Metabolic transitions within founding and niche families.
