# Supplementary material for: Innovation and tinkering in the evolution of oxidases
Source: Protein Sci. 2022 Apr 26;31(5):e4310. doi: 10.1002/pro.4310 (PMC9040561; doi:10.1002/pro.4310)
Supplement: Supplementary file 1 — Appendix S1 Supporting Information. [file PRO-31-e4310-s001.pdf]

Supplementary Information

Identification of O_2-enzyme families

To identify all known O_2-enzyme families, we searched the KEGG database[1] for all reactions that involve dioxygen as a substrate. For every such reaction, we extracted the enzyme(s) known to catalyze it. Overall, we identified 730 enzymes (individual EC classes), mostly belonging to two classes: dioxygenases (EC 1.13.-.-) and monooxygenases (1.14.-.-). We extracted sequences associated with these enzymes from the ExPASy Enzyme database[3] of characterized enzymes. In total, 537/730 enzymes were represented by at least one sequence. The remaining 193 enzymes were either not represented by a sequence in the ExPASy Enzyme database. To identify the catalytic (O_2-related) and auxiliary (O_2-unrelated) domains to these enzymes, we assigned the Pfam families with HMMsearch[2] (with gathering threshold) these sequences and combined with analysis of available structures, we identified the domain universally present in all related enzymes as being the catalytic one. In ambiguous cases, manual assignment based on available literature has been performed. Finally, we cross-validated the EC to Pfam mappings using ECDomainMiner[4] that assigns catalytic domains to EC classes. Overall, we mapped the 537 O_2-enzymes to 136 Pfam families (a given EC number, *i.e.*, enzyme class, might be catalyzed by multiple families; conversely, a given Pfam family may include dozens of EC numbers; **Supplementary Table 1**).

Identification of niche *versus* founding functionalities

In 81 out of 136 Pfam families, the oxygen-utilizing or producing activity is assigned to nearly all family members (O_2 *founding* function); thus, by parsimony, the ancestor of the family was likely also an O_2 enzyme. In 55 families, non-O_2 enzyme function(s) are dominant. Therefore the ancestor of the family was likely an O_2 independent enzyme/protein. We identified the non-O_2 associated EC classes included in the mapped Pfam families using the ECDomainMiner database[5] querying for EC classes associated with the given Pfam family. A typical example is families comprising enzymes that perform the same or similar reactions with alternative electron acceptors such as dehydrogenases that use $NAD(P)^+$ that are in the same Pfam family with oxidases that utilize O_2 (Ref. [6]); for example, vanillyl alcohol oxidase and 4-methylphenol dehydrogenase that share significant sequence identity (>30%)[7]. For each *niche* family, we selected the most abundant enzyme class as the family ancestor (**Supplementary Table 1**).

Metabolic reactions emergence analysis

For each EC class, the reactions represented by these EC classes have been retrieved from the KEGG database. We used the annotation from KEGG to assign whether a given reaction is a part of a catabolic or anabolic metabolic pathway. For anabolic reactions, i.e., reactions whose primary metabolic role is the biosynthesis of a particular metabolite, all reactions that produce this compound are also identified in KEGG. Conversely, for catabolic reactions that degrade a given metabolite, we retrieved all O_2-independent reactions that catabolize the metabolite. In ambiguous cases, where the nature of the reaction has not been assigned, we assigned them

to the "Unknown" category—the complete list of reactions assigned to metabolic roles can be found in **Supplementary Table 2**.

Example enzyme analysis

To exemplify the analysis, the annotation pipeline of persulfide dioxygenase to all three levels of innovation is provided. Persulfide dioxygenase (EC 1.13.11.18) belongs to the Metallo-β-lactamase family (Pfam: Lactamase_B). The Metallo-β-lactamase is a family dominated by hydrolytic enzymes with broad specificity, including eponymous B-lactamases involved in bacterial resistance to β-lactam antibiotics, as well as various thiolesterases. Therefore, by parsimony, the ancestor of β-lactamase family was likely an enzyme with hydrolytic function, making an oxygen-dependent member of the family - persulfide dioxygenase - a *niche* oxygen enzyme that can be classified into category 1.2 – "transitions from non-redox enzymes" of chemistry transition (from hydrolase to oxidase trajectory, **Supplementary Table 1**).

Persulfide dioxygenase performs a following metabolic reaction (KEGG reaction ID: R08678): S-sulfanylglutathione + O_2 + H_2O ⇆ glutathione + sulfite + 2 H^+;
Where S-sulfanylglutathione is being oxidized to glutathione and sulfite in a sulfide catabolic pathway. Without the use of oxygen, S-sulfanylglutathione can be alternatively degraded in an O_2-independent reaction catalyzed by an enzyme thiosulfate sulfurtransferase (EC 2.8.1.1) to thiolsulfate. Since the degradation products in O_2 and O_2-free reactions differ (glutathione+sulfite or glutathione+thiosulfate respectively), persulfide oxidase can be classified to metabolic category 2.2 of metabolic transitions – "New transformations" (**Supplementary Table 2**).

As suggested before, persulfide oxidase likely evolved from a hydrolase from the Metallo-β-lactamase family. The hallmark of hydrolases from this family is a dinuclear metal active site. The persulfide oxidase possesses a single metal ion in the active site, and the second metal site is likely replaced by a water molecule. Additionally, the oxidase metal side is occupied by an iron ion instead of zinc in hydrolases from this family (see main text).

Based on the described pipeline, every enzyme can be analyzed accordingly.

References

1. Kanehisa, M. KEGG: Kyoto Encyclopedia of Genes and Genomes. *Nucleic Acids Res.* **28**, 27–30 (2000).
2. Eddy, S. R. Accelerated profile HMM searches. *PLoS Comput. Biol.* **7**, e1002195 (2011).
3. Bairoch, A. The ENZYME database in 2000. *Nucleic Acids Res.* **28**, 304–305 (2000).
4. Alborzi, S. Z., Devignes, M. D. & Ritchie, D. W. ECDomainMiner: Discovering hidden associations between enzyme commission numbers and Pfam domains. *BMC Bioinformatics* **18**, 107 (2017).
5. Alborzi, S. Z., Devignes, M. D. & Ritchie, D. W. ECDomainMiner: Discovering hidden associations between enzyme commission numbers and Pfam domains. *BMC Bioinformatics* **18**, (2017).
6. Raymond, J. & Blankenship, R. E. Biosynthetic pathways, gene replacement and the

antiquity of life. *Geobiology* **2**, 199–203 (2004).
7. Gygli, G., Lucas, M. F., Guallar, V. & van Berkel, W. J. H. The ins and outs of vanillyl alcohol oxidase: Identification of ligand migration paths. *PLoS Comput. Biol.* **13**, e1005787 (2017).
